# Comparing prostatic artery embolization to surgical and minimally invasive procedures for the treatment of benign prostatic hyperplasia: a systematic review and meta-analysis

**DOI:** 10.1186/s12894-023-01397-1

**Published:** 2024-01-28

**Authors:** Rachel Altman, Roseanne Ferreira, Camilo Barragan, Naeem Bhojani, Katherine Lajkosz, Kevin C. Zorn, Bilal Chughtai, Ganesan Annamalai, Dean S. Elterman

**Affiliations:** 1 Division of Urology, Department of Surgery, University Health Network, Toronto, ON Canada; 2 Department of Vascular and Interventional Radiology, University Health Network, Mount Sinai Hospital, Toronto, ON Canada; 3 Department of Surgery, University of Montreal Hospital Center, Montreal, QC Canada; 4 Department of Biostatistics, University Health Network/Princess Margaret Cancer Centre, Toronto, ON Canada; 5 Department of Urology, Weill Cornell Medical College, New York, NY USA; 6 Department of Vascular and Interventional Radiology, University Health Network, University of Toronto, Toronto, ON Canada

**Keywords:** Benign prostatic hyperplasia, Transurethral resection of the prostate, Lower urinary tract symptoms, Open simple prostatectomy, Prostatic artery embolization, Minimally invasive

## Abstract

**Background:**

To summarize current evidence to report a comparative systematic review and meta-analysis of prostatic artery embolization (PAE) with transurethral resection of the prostate (TURP) and open simple prostatectomy (OSP) for the treatment of benign prostatic hyperplasia (BPH).

**Methods:**

A systematic literature search was performed to identify studies published from inception until August 2021. The search terms used were (prostate embolization OR prostatic embolization) AND (prostatic hyperplasia OR prostatic obstruction) as well as the abbreviations of PAE and BPH. Risk of bias was assessed using the Cochrane Risk of Bias tool for randomized controlled trials (RCTs) and the Risk of Bias in Non-randomized Studies—of Interventions (ROBINS-I) tool for observational studies. Random-effects meta-analysis was performed using Revman 5.4.

**Results:**

Seven studies were included with 810 patients: five RCTs and one observational study compared PAE with TURP, and one observational study compared PAE with OSP. The included studies had considerable risk of bias concerns. TURP and OSP were associated with more statistically significant improvements in urodynamic measures and BPH symptoms compared to PAE. However, PAE seems to significantly improve erectile dysfunction compared to OSP and improve other outcome measures compared to TURP, although not significantly. PAE appeared to reduce adverse events and report more minor complications compared with TURP and OSP, but it is unclear whether PAE is more effective in the long-term.

**Conclusion:**

PAE is an emerging treatment option for patients with symptomatic BPH who cannot undergo surgery or have undergone failed medical therapy. Overall, PAE groups reported fewer adverse events. Future ongoing and longer-term studies are needed to provide better insight into the benefit of PAE compared to other treatment options.

**Supplementary Information:**

The online version contains supplementary material available at 10.1186/s12894-023-01397-1.

## Background

Benign prostatic hyperplasia (BPH) is the non-malignant enlargement of the prostate that often leads to lower urinary tract symptoms (LUTS) [1] BPH can put severe pressure on the urethra and bladder, which impairs bladder voiding [1]. LUTS secondary to BPH can include a slow urinary stream, hesitancy, straining and can lead to serious complications including urinary retention, infection, and renal dysfunction [2]. As males age, the prevalence of BPH increases, accounting for 60% of LUTS in males aged 50–60-years-old. Approximately 50% of men will develop BPH above 50 years of age, with an increase to 80% by age 80 [3, 4]. Although age is a main risk factor for BPH, other contributing factors might be metabolic syndrome, including hypertension and diabetes [3]. Some males with BPH are asymptomatic and are not affected by this diagnosis; however, when symptomatic, this condition can critically impact an individuals’ quality of life. Moderate to severe symptoms often require minimally invasive procedures to remove excess tissue or shrink the prostate gland [1, 3]. Medications such as alpha-blockers and 5-alpha-reductase inhibitors are common primary treatment options for BPH [5]. Patients with symptoms refractory to these medical therapies may be subsequently treated with surgical procedures, such as transurethral resection of the prostate (TURP), which is recognized as the gold-standard [4]. There are risks associated with the TURP procedure due to its surgical nature such as retrograde ejaculation, and patient outcomes are shown to be variable [1, 4]. In addition, open simple prostatectomy (OSP) is an alternative surgical technique to TURP used to treat LUTS secondary to BPH [6]. OSP is thought to be more advantageous for men with moderate to severe LUTS and a prostate size that exceeds 80 mL [7, 8]. Notably, this technique may also be preferred when treating concomitant bladder stones as these can be extracted simultaneously [9]. However, OSP has been associated with notable perioperative morbidity including bleeding complications, necessitating the introduction of newer and minimally invasive treatments [6].

Minimally invasive options for LUTS secondary to BPH have emerged, such as prostatic artery embolization (PAE) [3]. PAE is an endovascular procedure where tiny particles are injected into the prostatic arteries to reduce blood flow to the prostate, causing it to shrink in size [10]. A systematic review was performed to evaluate the available evidence on the clinical outcomes of PAE as it compares to other standard surgical interventions, namely TURP and OSP.

## Methods

### Search strategy and selection criteria

We performed a systematic literature search to identify relevant studies published from inception until August 2021. The search strategies were developed using controlled vocabulary and relevant keywords to prostatic artery embolization for BPH. The search terms used were (prostate embolization OR prostatic embolization) AND (prostatic hyperplasia OR prostatic obstruction) as well as the abbreviations of PAE and BPH. The primary search was conducted in PubMed, supplemented by searches in the Medline database and the Cochrane Central Register of Controlled Trials.

We included studies if they were (1) English-language full-text publications; (2) randomized controlled trials (RCTs) or prospective comparative non-randomized studies of participants with BPH; (3) included PAE interventions using any particle type, size, and embolization technique; (4) performed PAE through the femoral or radial artery. Acceptable comparators were surgical or minimally invasive procedures for BPH. We excluded (1) narrative reviews, retrospective studies, systematic reviews and meta-analyses, abstracts, editorials, case reports and commentaries; (2) non-comparative studies; (3) non-human studies.

### Data abstraction and risk of bias assessment

Evidence included in this systematic review was acquired in accordance with the Preferred Reporting Items for Systematic Review and Meta-analysis (PRISMA) guidelines [11]. Two independent reviewers extracted data from included studies, using a standardized form. Any discrepancies were resolved through discussion or, if necessary, adjudicated by a third reviewer (senior author). We evaluated the risk of bias using the Cochrane Collaboration [12] for randomized controlled trials and Risk Of Bias In Non-randomized Studies of Interventions (ROBINS-I) [13] tools for non-randomized studies for each individual study.

### Data synthesis and statistical analysis

#### Outcome measures

The clinical outcomes of interest were prostate-specific antigen (PSA) levels, prostate volume (PV), post-void residual urine volume (PVR), and peak urinary flow rate (Qmax). Self-reported scores for international prostate symptom score (IPSS), health-related quality of life (QOL), and erectile function (IIEF) were included. A seven-question scale was used to rate international prostate symptom score (IPSS) urinary symptoms as mild, moderate, or severe [14]. The IPSS health-related quality of life (IPSS-QOL) scale was used and ranges from 1 (delighted) to 6 (terrible), with a decrease in this score indicating improved QOL [14]. Sexual outcomes were measured by International Index of Erectile Function (IIEF-5), a five-question scale to measure the degree of erectile dysfunction with a higher score indicating less erectile dysfunction [15]. Secondary outcome measures included adverse events as reported by each study.

#### Synthesis

Studies were compared to evaluate for clinical and methodological heterogeneity and a meta-analysis was planned. Comparative analyses utilized the random-effects model through RevMan 5.4 software. A meta-analysis was performed by grouping studies based on their study design. A two-tailed statistical test, with a 0.05 probability threshold for type 1 error, determined the statistical significance of the combined results. For continuous variables, the inverse variance method was applied, presenting treatment effects as mean differences (MD) alongside standard deviation (SD) of mean difference and 95% confidence intervals (CIs). To measure the extent of variance attributable to heterogeneity rather than chance, Higgins I^2^ statistics were employed.

For outcomes with high clinical heterogeneity between study groups (different control treatments) or less than 2 studies per outcome, a meta-analysis was not performed. A summary of results were reported descriptively for these studies.

#### Assessing certainty of evidence

Based on the *Grading of Recommendations Assessment, Development, and Evaluation* (GRADE) methodology [16–18], the quality of evidence for each outcome was evaluated considering the five domains: risk of bias, inconsistency, indirectness, imprecision, and publication bias. The body of evidence was categorized from high, moderate, low and very low certainty levels.

## Results

### Description of evidence

**Fig. 1:**
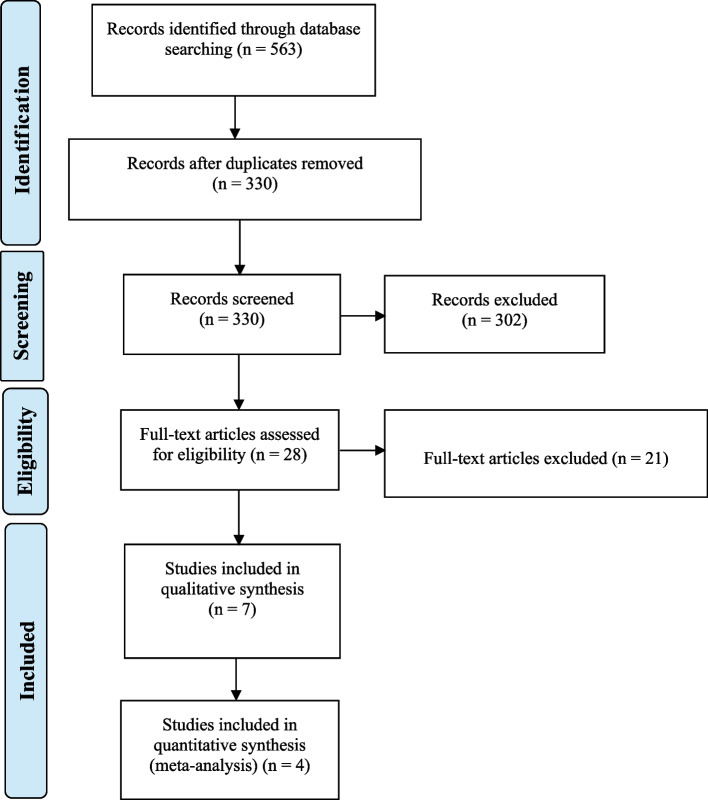
PRISMA diagram outlining article selection for review

The literature search identified 563 records, of which seven publications with (810 patients) including five RCTs [19–23] and two non-randomized observational comparative studies [24, 25] met eligibility criteria. Six of the included studies compared PAE and TURP [19–24] and one non-randomized study compared PAE with OSP [25]. Table 1 presents the study characteristics of the seven included studies, following the PRISMA diagram (Fig. 1).

**Table 1:** Study characteristics

Author, Year Country	Study Design	N	Inclusion Criteria	Exclusion Criteria	Mean Age ± SD	Interventions	Procedure Time (min)	Follow Up Time
Abt et al. 2021 [19] Switzerland	RCT	Total: 81 PAE: 34 TURP: 47	Males ≥ 40 yrs; TURP indicated after medical treatment or refusal of medical treatment; PV 25–80 mL; IPSS ≥ 8; QOL ≥ 3; Qmax < 12 mL/s or urinary retention	Severe arterial disease; acontractile detrusor; neurogenic lower urinary tract dysfunction; urethral stenosis; bladder diverticulum; bladder stone; allergy to intravenous contrast media, contraindication for MRI, prostate cancer; renal failure	PAE: 65.7 ± 9.3 TURP: 66.1 ± 9.8	PAE: performed bilaterally or unilaterally under local anesthesia; 250-400μm microspheres TURP: Monopolar TURP under spinal or general anaesthesia	PAE: 122.2 (25.8) TURP: 69.5 (22.5)	3, 6, 12, 24 mo
Carnevale et al. 2016 [20] Brazil	RCT	Total: 45 PAE: 15 PErFecTED PAE: 15 TURP: 15	Males ≥ 45 yrs; IPSS > 19; symptoms refractory to medical treatment for ≥ 6 mo; Qmax ≤ 12; PV 30–90 ml	Renal failure; bladder calculi or diverticula; suspected prostate cancer; urethral stenosis, neurogenic bladder disorders	Original PAE: 63.5 ± 8.7 PErFecTED: 60.4 ± 5.2 TURP: 66.4 ± 5.6	PAE: performed bilaterally under local anesthesia; 300-500μm microspheres (original PAE and PErFecTED PAE) TURP: Monopolar TURP under spinal anesthesia	Original PAE: 144.8 ± 50.1 PErFecTED PAE: 147.5 ± 30.4 TURP: 61.7 ± 17.0	12 mo
Gao et al. 2014 [21] China	RCT	Total: 114 PAE: 57 TURP: 57	IPSS > 7; failed medical therapy with 2 wks washout period; PV 20–100 mL, Qmax < 15 mL/s	Detrusor hyperactivity or hypocontractility; urethral stricture; prostate cancer; diabetes mellitus; previous prostate, bladder neck, or urethral surgery	PAE: 67.7 ± 8.7 TURP: 66.4 ± 7.8	PAE: performed bilaterally or unilaterally under local anesthesia; 355-500μm TURP: Bipolar TURP under epidural anesthesia	PAE: 89.7 ± 17.1 TURP: 83.5 ± 17.5	1, 3, 6, 12, 24 mo
Insausti et al. 2020 [22] Spain	RCT	Total: 45 PAE: 23 TURP: 22	Males > 60 yrs; BPH-related LUTS refractory to medical treatment for ≥ 6 mo or could not receive medical; IPSS ≥ 8; QOL ≥ 3, Qmax ≤ 10 ml/s or urinary retention	Severe arterial disease; non-visualization of the prostatic artery; urethral stenosis; detrusor failure or neurogenic bladder; GFR < 30 ml/min, prostate cancer	PAE: 72.4 ± 6.2 TURP: 71.8 ± 5.5	PAE: performed bilaterally under local anesthesia; 300-500μm microspheres TURP: Bipolar TURP under spinal or general anesthesia	PAE: 138.7 (51.9) TURP: 70.2 (21.1)	3, 6, 12 mo
Radwan et al. 2020 [23] Egypt	RCT	Total: 60 PAE: 20 M-TURP: 20 B-TURP: 20	IPSS 8–35; average flow ≤ 10 ml/s, PV < 100 ml	Elevated kidney functions; allergy to IV contrast medial; prostate cancer; history of prostatic or urethral operations; signs of decompensated bladder; urinary tract infection	PAE: 63 TURP: 63	PAE: performed bilaterally under local anesthetic; 6 microspheres TURP: Monopolar or bipolar TURP	PAE: 89 M-TURP: 59 B-TURP: 68	6 mo
Ray et al. 2018 [24] United Kingdom	Observational study	Total: 305 PAE: 216 TURP: 90	Males with LUTS; fluent in English	Not stated by the study	PAE: 66 ± 7.4 TURP: 70 ± 7.5	PAE: PVA microspheres TURP: Monopolar and bipolar TURP	PAE: 144 (51) TURP: NR	1, 3, 6, 12 mo
Russo et al. 2015 [25] Italy, Russia	Observational study	Total: 160 PAE: 80 OSP: 80	IPSS > 12; PSA level < 4 ng/ml or 4–10 ng/ml; negative prostate biopsy; PV > 80 cm^3^; Qmax < 15 mL/s	Neurogenic bladder dysfunction and/or sphincter decompensation, coagulation disorders; chronic kidney disease, previous surgical or medical treatment with 5-alpha reductase inhibitors; life expectancy < 2 yrs; bladder stones; catheter or acute retention of urine in the last 4 wks	PAE: 67.0 ± 5.72 TURP: 68.4 ± 6.13	PAE: 300-500μm microspheres OSP: Suprapubic transvesical open prostatectomy	PAE: 84 (68–101)^a^ OSP: 57 (42–74)^a^	1, 6, 12 mo

Data for procedure time reported as mean (standard deviation) or mean ± standard deviation unless otherwise specified

*Abbreviations:* *RCT* randomized controlled trial, *SD* standard deviation, *TURP* transurethral resection of the prostate, *BPH* benign prostatic hyperplasia, *OSP* open simple prostatectomy, *PAE* prostatic artery embolization, *MRI* magnetic resonance imaging, *PV* prostate volume, *GFR* glomerular filtration rate, *IPSS* International Prostate Symptom Score, *LUTS* lower urinary tract symptoms, *PErFecTED* proximal embolization first then embolize distal method of prostatic artery embolization, *M-TURP* monopolar transurethral resection of the prostate, *B-TURP* bipolar transurethral resection of the prostate, *PVA* polyvinyl alcohol *Qmax* peak urinary flow rate, *QOL* quality of life, *NR* not reported

^a^Data reported as median (range)

A detailed summary of functional outcome measures reported by each study is shown in the Additional file 1 (Supplementary Tables S1-S7). IIEF-5 [15] was used by all studies except for Insausti et al. [22] and Gao et al. [21] which did not assess erectile function in their studies. Three of the six included studies presented one year follow-up times. Abt et al. [19] and Gao et al. [21] had the longest follow-up of the included studies, with 24-month data comparing PAE with TURP. Baseline and 12-month follow-up outcomes are summarized for all studies except for Abt et al. [19] which did not report 12-month outcomes for the PV outcome measure only.

### Risk of bias assessment of included studies

**Fig. 2:**
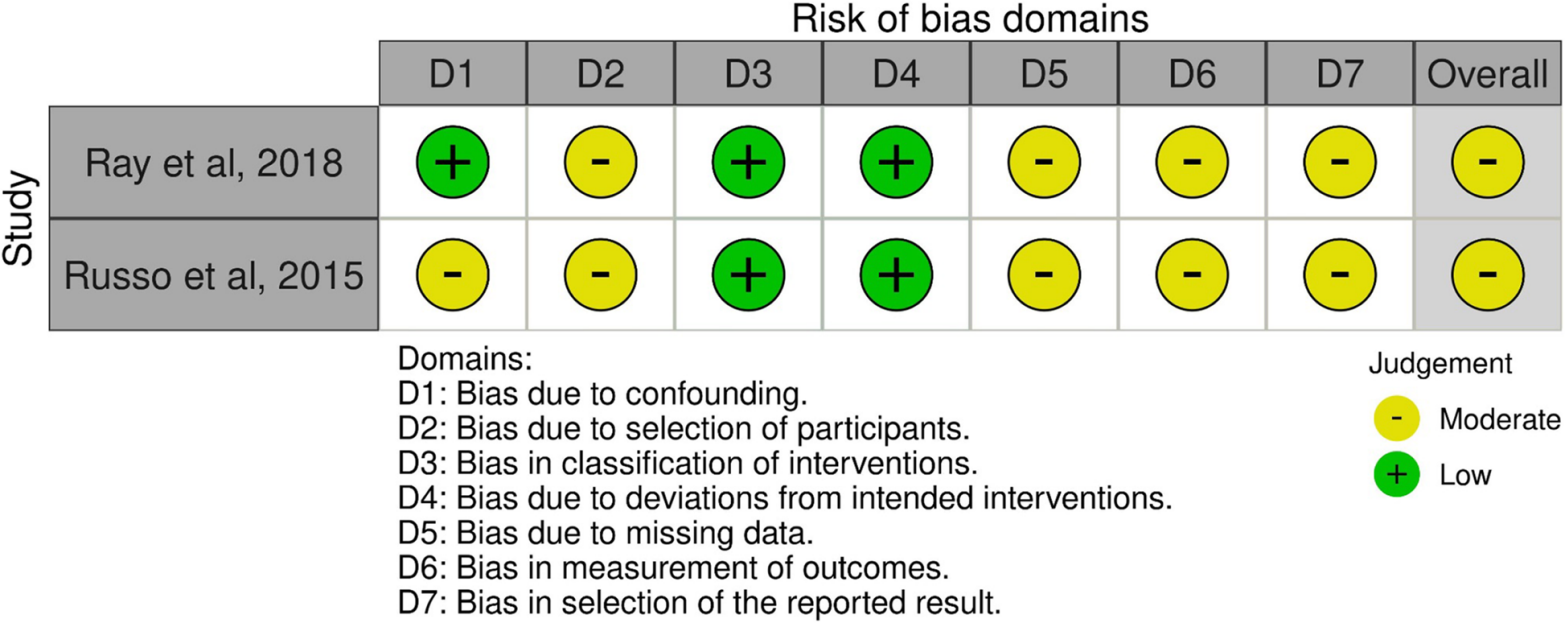
Risk of bias among non-randomized trials (ROBINS-I Tool). Abbreviation: ROBINS-I, Risk of Bias in Non-randomized Studies—of Interventions

The assessment of bias varied across studies, despite studies addressing similar outcomes. Summaries of the risk of bias evaluations, conducted using the ROBINS-I and the Cochrane Collaboration's tool, are presented in (Fig. 2) and Table 2. Most randomized studies had a high overall risk of bias.

**Table 2:** Risk of bias among randomized controlled trials (Cochrane Collaboration)

Author, Year	Random Sequence Generation	Allocation Concealment	Blinding of Participants and Personnel	Blinding of outcome assessment	Incomplete Outcome Data	Selective Reporting	Other Bias
Abt et al. 2021 [9]	Low	Low	Low	High	Low	Low	High
Carnevale et al. 2016 [10]	Unclear	Unclear	Unclear	High	Low	Low	High
Gao et al. 2014 [11]	Low	High	Low	High	Low	High	High
Insausti et al. 2020 [12]	Low	Unclear	Low	High	Low	Low	High
Radwan et al. 2020 [23]	Unclear	Low	High	High	Low	High	Low

### Baseline vs. 12-month outcomes for PAE vs. TURP

#### IPSS

**Fig. 3:**
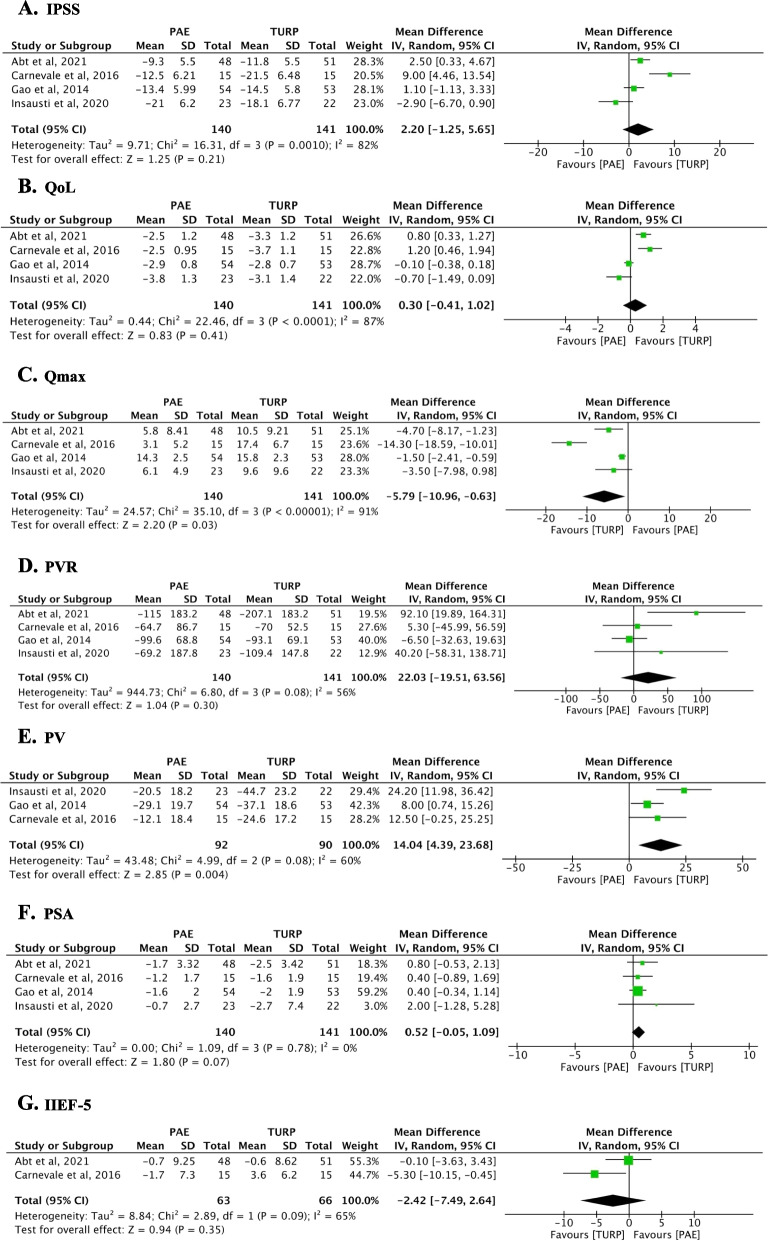
Forest plots showing the mean difference between 12 month and baseline outcome variables between prostatic artery embolization (PAE) vs. transurethral resection of the prostate (TURP). **A** IPSS; **B** QoL; **C** Qmax (ml/s); **D** PVR (ml); **E** PV (ml); **F**. PSA (ng/ml); **G** IIEF-5. Abbreviations: IPSS, International Prostate Symptom Score; Qol, quality of life; IIEF-5, International Index of Erectile Function 5; Qmax (ml/s), maximum flow rate; PV, prostate volume; PVR (ml), postvoid residual volume; PSA (ng/ml), prostate-specific antigen; PAE, prostatic artery embolization; TURP, transurethral resection of the prostate

Across five RCTs examining IPSS scores, our meta-analysis did not reveal a significant difference between PAE and TURP (*n* = 441; weighted mean difference [WMD]: 2.20; 95% CI: − 1.25 to 5.65; *p* = 0.21), with substantial heterogeneity detected (I^2^ = 82%, *p* < 0.0001) (Fig. 3A). At the same time point, Ray and colleagues showed a mean decrease in IPSS of -10.8 ± 25.8 for PAE and -15.2 ± 25.8 for TURP, indicating no significant overall difference between the two procedures [24]. Radwan et al. [23] also observed a greater reduction of IPSS in the TURP group at 6 months (-14 ± 5.23 mean reduction at the PAE group vs -18 ± 5.04 in the TURP group). For detailed comparisons between baseline, 3, 6, 12 and 24 months please see (Additional file 1: Table S1).

#### Quality of Life (QoL)

Four studies assessed QoL outcomes, showing no significant distinction between the groups (*n* = 281; WMD: 0.30; 95% CI: − 0.41 to 1.02; *p* = 0.41), though significant heterogeneity was present (I^2^ = 87%, *p* < 0.0001) (Fig. 3B). Similarly, the non-randomized study by Ray et al. [24] reported a mean QoL improvement of -2.6 ± 1.4 for PAE versus -3.4 ± 1.4 for TURP from baseline to 12 months, consistent with our findings of no significant difference in QoL between treatments.

#### Maximum urinary flow rate (Qmax)

Functional outcome analysis for Qmax from five randomized trials indicated a significant improvement in TURP compared to PAE (*n* = 281; WMD: − 5.79; 95% CI: − 10.96 to − 0.63; *p* = 0.03). This analysis, however, revealed substantial heterogeneity (I^2^ = 91%, *p* < 0.00001), as illustrated in (Fig. 3C). Complementing these findings, Radwan et al. [23] reported Qmax enhancements at 6 months that were consistent with the 12-month follow-up, again favoring TURP (PAE group mean difference: + 4.7 ± 8.2 ml/s versus TURP group: + 14.9 ± 9.3 ml/s). At the one-year mark, Ray et al. [24] also documented improvements, with PAE patients showing a mean Qmax increase of 4.4 ± 4.7 ml/s and those undergoing TURP, an increase of 8.6 ± 6.3 ml/s.

#### Post-void residual volume (PVR)

For PVR, data pooled from four RCTs did not show a significant difference between PAE and TURP (*n* = 281; WMD: 22.03; 95% CI: − 19.51 to 63.56; *p* = 0.30), with moderate heterogeneity (I^2^ = 56%, *p* = 0.08) (Fig. 3D). The PVR change reported by Ray and colleagues [24] was -40.4 ± 136.0 ml for PAE and -79.8 ± 202.4 ml for TURP, consistent with the meta-analysis result of no significant difference in PVR reduction between the procedures.

#### Prostate volume (PV)

Analysis of PV from three RCTs revealed a significant reduction favoring TURP (*n* = 182; WMD: 14.04; 95% CI: 4.39 to 23.68; *p* = 0.004), with moderate heterogeneity (I^2^ = 60%, *p* = 0.08) (Fig. 3E). At 6 months, Radwan’s trial reported reduction in PV was consistent with meta-analysis 12 month results and favoured TURP [23]. PV in the PAE group reduced 11 ± 22.9ml vs 34 ± 26.6ml from the TURP group.

#### Prostate-specific antigen (PSA)

Our meta-analysis included four RCTs which reported PSA levels, showing no significant difference between PAE and TURP (n = 2811; WMD: 0.52; 95% CI: − 1.05 to 2.09; *p* = 0.07), with no heterogeneity (I^2^ = 0%, *p* = 0.78) (Fig. 3F).

#### International index of erectile function-5 (IIEF-5)

The IIEF-5 scores reported by two trials did not indicate a significant difference between PAE and TURP (*n* = 129; WMD: − 2.42; 95% CI: − 7.49 to 2.64; *p* = 0.35), despite significant heterogeneity (I^2^ = 65%, *p* = 0.09) (Fig. 3G). In Ray et al. [24], the IIEF-5 score improved by 1 ± 7 point for PAE and reduced by 0.2 ± 6.7 points for TURP, showing comparable sexual function outcomes post-treatment for both treatment groups.

### Critical appraisal of the evidence

The GRADE assessment of evidence provided by the included studies “very low to low” across all outcomes, indicating that “further research is very likely to have an important impact on our confidence in the estimate of effect and is likely to change the estimate” as well as “any estimate of the effect is very uncertain” [16–18].

### Prostatic artery embolization (PAE) vs. open simple prostatectomy (OSP)

The study by Russo et al. [25] was the only eligible study offering a comparative analysis between PAE and OSP. Table 3 highlights that although initial IPSS readings were similar for both treatments, OSP demonstrated a significantly greater reduction at both 6 months (PAE 11.4 vs. OSP 4.9; *p* < 0.01) and 12 months (PAE 10.4 vs. OSP 4.3; *p* < 0.01), signaling better symptom relief in this cohort. Quality of life also favored OSP with a notable improvement at 12 months (PAE 2.8 vs. OSP 0.7; *p* < 0.01), despite comparable baseline values (Table 4). In contrast, PAE patients maintained better erectile function over time, with significant differences seen at 6 months (PAE 15.5 vs. OSP 10.7; *p* < 0.01) and 12 months (PAE 15.1 vs. OSP 10.9; *p* < 0.01) (Table 5). OSP patients experienced a superior peak urinary flow rate with higher measurements at both 6 months (PAE 16.2 mL/s vs. OSP 24.5 mL/s; *p* < 0.01) and 12 months (PAE 16.9 mL/s vs. OSP 23.8 mL/s; *p* < 0.01) (Table 6).

**Table 3:** International prostatic symptom score (IPSS) for prostatic artery embolization (PAE) vs. open simple prostatectomy (OSP)

Mean IPSS (95% CI): Baseline				
**Study**	**N**	**PAE**	**OSP**	***P* Value**
Russo et al. 2015 [14]	PAE: 80 OSP: 80	24.0 (22.7–25.3)	23.4 (22.3–24.4)	.53
**Mean IPSS (95% CI): 6 Months**				
Russo et al. 2015 [14]	PAE: 80 OSP: 80	11.4 (10.7–12.0)	4.9 (4.2–5.7)	< .01
**Mean IPSS (95% CI): 12 Months**				
Russo et al. 2015 [14]	PAE: 80 OSP: 80	10.4 (9.4–11.4)	4.3 (3.6–5.0)	< .01

*Abbreviations: CI* confidence interval

**Table 4:** International prostate symptom score-quality of life (IPSS-QoL) for prostatic artery embolization (PAE) vs. open simple prostatectomy (OSP)

Mean IPSS-QoL (95% CI): Baseline				
**Study**	**N**	**PAE**	**OSP**	***P* Value**
Russo et al. 2015 [14]	PAE: 80 OSP: 80	4.4 (4.2–4.6)	4.1 (3.9–4.3)	.1
**Mean IPSS (95% CI): 12 Months**				
Russo et al. 2015 [14]	PAE: 80 OSP: 80	2.8 (2.6–3.0)	0.7 (0.6–0.9)	< .01

Abbreviations: *CI* confidence interval

**Table 5:** International index of erectile function (IIEF) for prostatic artery embolization (PAE) vs. open simple prostatectomy (OSP)

Mean IIEF-5 (95% CI): Baseline				
**Study**	**N**	**PAE**	**OSP**	***P* Value**
Russo et al. 2015 [14]	PAE: 80 OSP: 80	14.5 (13.4–15.5)	15.1 (13.8–16.4)	.56
**Mean IIEF-5 (95% CI): 6 Months**				
Russo et al. 2015 [14]	PAE: 80 OSP: 80	15.5 (14.4–16.7)	10.7 (9.0–12.4)	< .01
**Mean IIEF-5 (95% CI): 12 Months**				
Russo et al. 2015 [14]	PAE: 80 OSP: 80	15.1 (14.0–16.2)	10.9 (9.2–12.6)	< .01

*Abbreviations*: *CI* confidence interval, *IIEF* International Index of Erectile Function, *OSP* open simple prostatectomy, *PAE* prostatic artery embolization

**Table 6:** Peak urinary flow rate (Qmax) for prostatic artery embolization (PAE) vs. open simple prostatectomy (OSP)

Mean Qmax, mL/s (95% CI): Baseline				
**Study**	**N**	**PAE**	**OSP**	***P* Value**
Russo et al. 205 [25]	PAE: 80 OSP: 80	7.3 (6.5–8.0)	7.9 (7.5–8.2)	.21
**Mean Qmax, mL/s (95% CI): 6 Months**				
Russo et al. 205 [25]	PAE: 80 OSP: 80	16.2 (15.2–17.2)	24.5 (23.3–25.7)	< .01
**Mean Qmax, mL/s (95% CI): 12 Months**				
Russo et al. 2015 [25]	PAE: 80 OSP: 80	16.9 (15.8–18.0)	23.8 (22.5–25.1)	< .01

*Abbreviations*: *CI* confidence interval

Table 7 shows OSP’s advantage in minimizing PVR with significantly lower volumes at 6 months (PAE 19.2 mL vs. OSP 4.3 mL; *p* < 0.01) and 12 months (PAE 18.4 mL vs. OSP 6.2 mL; *p* < 0.01). Furthermore, Table 8 illustrates a reduction in PSA levels for the OSP group at both 6 and 12 months (mean PSA at 12 months: PAE 2.1 ng/mL vs. OSP 1.3 ng/mL; *p* < 0.01), pointing to a potentially more effective impact on this parameter by OSP. Lastly, there was no information regarding pre-post procedural changes in PV between treatment arms.

**Table 7:** Post-void residual volume (PVR) for prostatic artery embolization (PAE) vs. open simple prostatectomy (OSP)

Mean PVR, mL (95% CI): Baseline				
**Study**	**N**	**PAE**	**OSP**	***P* Value**
Russo et al. 2015 [25]	PAE: 80 OSP: 80	64.3 (57.0–71.5)	65.0 (51.0–78.9)	.95
**Mean PVR, mL (95% CI): 6 Months**				
Russo et al. 2015 [25]	PAE: 80 OSP: 80	19.2 (17.0–21.4)	4.3 (3.4–5.2)	< .01
**Mean PVR, mL (95% CI): 12 Months**				
Russo et al. 2015 [25]	PAE: 80 OSP: 80	18.4 (16.3–20.5)	6.2 (5.2–7.1)	< .01

*Abbreviations*: *CI* confidence interval, *NA* not applicable, *NR* not reported

**Table 8:** Prostate-specific antigen (PSA) levels for prostatic artery embolization (PAE) vs. open simple prostatectomy (OSP)

Mean PSA, ng/mL (95% CI): Baseline				
**Study**	**N**	**PAE**	**OSP**	***P* Value**
Russo et al. 2015 [25]	PAE: 80 OSP: 80	3.6 (3.2–4.0)	4.2 (3.8–4.6)	.1
**Mean PVR, mL (95% CI): 6 Months**				
Russo et al. 2015 [25]	PAE: 80 OSP: 80	2.4 (2.2–2.6)	1.4 (0.9–1.9)	< .01
**Mean PVR, mL (95% CI): 12 Months**				
Russo et al. 2015 [25]	PAE: 80 OSP: 80	2.1 (1.9–2.3)	1.3 (1.0–1.7)	< .01

*Abbreviations*: *CI* confidence interval

### Adverse events

Table 9 displays the adverse events categorized by Clavien-Dindo Grade in the included studies comparing PAE and TURP. The most common adverse event reported by all included studies comparing PAE and TURP was hematuria followed by urinary infection which was reported by all included studies except for Carnevale et al. [20] (Additional file 1: Table S8). Overall, 260 adverse events were reported in the PAE groups compared to 310 in the TURP groups (Additional file 1: Table S8). In all of the studies that reported it, urinary incontinence and ejaculatory disorders were higher in the TURP group compared to PAE except for one study by Ray et al. [24] Among the included studies comparing PAE with TURP, mainly minor (Clavien Grade I and II) adverse events were reported in the PAE group, compared to some major (Clavien Grade III and IV) events reported in the TURP group. In all of the included studies except for Gao et al. [21], TURP patients experienced more major complications than PAE patients, with PAE patients reporting none in two studies [20, 22].

**Table 9:** Adverse events for prostatic artery embolization (PAE) vs transurethral resection of the prostate (TURP) classified by Clavien-Dindo Grade

**Adverse Event**	**Abt et al. 2021** [19]		**Carnevale et al. 2016** [20]			**Gao et al. 2014** [21]		**Insausti et al. 2020** [22]		**Radwan et al. 2020** [23]			**Ray et al. 2018** [24]	
	**PAE** **n (%)**	**TURP** **n (%)**	**Original PAE** **n (%)**	**PErFecTED PAE** **n (%)**	**TURP** **n (%)**	**PAE** **n (%)**	**TURP** **n (%)**	**PAE** **n (%)**	**TURP** **n (%)**	**PAE** **n (%)**	**M-TURP** **n (%)**	**B-TURP** **n (%)**	**PAE** **n (%)**	**TURP** **n (%)**
Clavien Grade I	22/34 (66%)	30/47 (64%)	NR	NR	NR	20/54 (37.0%)	8/53 (15.1%)	6/23 (26.7%)	12/22 (53.2%)	NR	NR	NR	NR	NR
Clavien Grade II	8/34 (24%)	10/47 (22%)	NR	NR	NR	2/54 (3.7%)	5/48 (10.4%)	17/23 (73.3%)	10/22 (44.7%)	NR	NR	NR	NR	NR
Clavien grade ≥ III	4/34 (11.8%)	10/47 (21.3%)	0/15 (0%)	0/15 (0%)	2/15 (13.3%)	8/54 (14.8%)	4/53 (7.5%)	0/23 (0%)	1/22 (4.5%)	NR	NR	NR	NR	NR

*Abbreviations*: *NR* not reported, *PErFecTED* Proximal embolization first then embolize distal method of PAE, *M-TURP* monopolar transurethral resection of the prostate, *B-TURP* bipolar transurethral resection of the prostate

When comparing PAE with OSP, PAE groups reported fewer adverse events overall [25]. The most common adverse events for both PAE and OSP were Clavien grade I with the PAE treatment group reporting less events than OSP. One case of urinary infection (Clavien II) was reported on the PAE group compared to 3 cases post OSP. No Clavien grade IIIa complications (complications that require an intervention performed under local anesthesia) [22] were found in the PAE group, but 3.8% were reported for the OSP group. Namely, 2 cases of urethral stricture and 1 case of urgency and incontinence were reported as major complications in the OSP group. This study by Russo et al. [25] reported 7 overall adverse events for the PAE group compared to 24 events in the OSP group (Table 10).

**Table 10:** Adverse events of studies comparing prostatic artery embolization (PAE) with open simple prostatectomy (OSP)

**Adverse Event**	**Russo et al. 2015** [25]	
	**PAE** **n (%)**	**Open Simple Prostatectomy** **n (%)**
Clavien grade I	6/80 (7.5%)	11/80 (13.8%)
Clavien grade II	1/80 (1.3%)	10/80 (12.5%)
Clavien grade IIIa	0/80 (0%)	3/80 (3.8%)

### Reintervention rate

The reintervention rates following PAE and TURP exhibit significant variability. PAE was associated with higher surgical reintervention rates. Notably, in the study by Abt et al. 2021 [19], 21% of patients undergoing initial PAE required subsequent TURP within two years for unsatisfactory outcomes, compared to a 7.8% surgical retreatment rate in the TURP cohort. Carnevale et al. [20] found that 13.3% of patients in the PAE group needed TURP reintervention post-PAE due to LUTS, whereas none in the TURP group required further intervention. Ray et al. [24] reported a total 20% reintervention for PAE (5% pre-12 months, 15% post-12 months), contrasting with 5.6% in TURP. Data on reintervention rates were not provided by Gao et al., Insausti et al., and Radwan et al. [21–23], and Russo et al. [25] did not address this aspect when comparing PAE versus OSP.

### Embolization particle size and radiation dose

From the embolization technique point of view, all PAEs were performed in a similar fashion. Most used embolic agents with particle size between 300 and 500 microns and similar embolic agent volume was administered. Abt et al. [19] used slightly smaller (250–400 microns), calibrated Embozene particles, but no significant differences in outcomes were reported. Two studies did not describe the amount of embolic used [22, 24] and one study did not record the size of the particles used [24]. Four out of the seven included studies reported radiation dose used during PAE, with two reporting the dose cumulatively (cGy/cm^2^) [21, 24] and two as mean dose (Gy/cm^2^) [19, 22]. Table 11 presents the embolization particle sizes and PVs before and after intervention as well as the radiation dose administered during PAE.

**Table 11:** Embolization particle size, prostate volumes (PV) and radiation dose

Study	PAE Patients (n)	Radiation Dose (SD) (Gy/cm^2^)	Bead size (microns)	Volume injected (ml)	Prostate Volume mean (ml)	PV after 6m (ml)	PV after 12m (ml)
Abt et al. 2021 [19]	34	176.5 (101.2)	250—400	1	51.2 (25 – 80) TAUS	NR	NR
Carnevale et al. 2016 [20]	30	NR	300—500	2	56.6 oPAE (45.7 – 67.5) – 66.2 PErFecTED (59.8 – 72.6) MRI	NR	50.9 oPAE (41.3 – 60.5) 50.0 PErFecTED (43.0 – 57.0)
Gao et al. 2014 [21]	57	11,305.1 (2671.5) (cGy/cm^**2**^**)**	355—500	2	64.7 (20 – 100) TRUS	36.3 (23.1 – 49.4)	35.6 (22.6 – 48.4)
Insausti et al. 2020 [22]	23	228.0 (61.6)	300—500	NR	60 (51.3 – 68.7) TAUS	37.7 (31.3 – 44.1)	39.5 (31.7 – 47.4)
Radwan et al. 2020 [23]	20	NR	300—500	2	60 (22.1) TRUS	49^b^	NR
Ray et al. 2018 [24]	216	17,892 (11,301–30905)^a^ (cGy/cm^**2**^**)**	NR	NR	101.2 (59.5 – 125.0) TRUS	NR	72.8 (Median 58.0)
Russo et al. 2015 [25]	80	NR	300—500	2	112.4 TRUS	NR	NR

Data are mean (standard deviation) unless otherwise indicated

*Abbreviations: TAUS* transabdominal ultrasound, *TRUS* transrectal ultrasound, *MRI* Magnetic Resonance Image, *NR* not reported *oPAE*, original PAE, *PErFecTED* Proximal Embolization First Then Embolize Distal, *SD* Standard Deviation

^a^Data for Ray et al. 2018 displayed as median (interquartile range)

^b^11cc reduction in prostate volume pre-post intervention

## Discussion

PAE is regarded as an effective, emerging treatment option for symptomatic BPH; however, its long-term clinical results are not as well established compared to other surgical procedures. This present meta-analysis highlights a comprehensive comparison of PAE and TURP across several clinical outcomes over a 12-month period. We found no significant difference in IPSS and QoL between PAE and TURP, indicating that both procedures are similarly effective in improving urinary symptoms and overall patient well-being. However, TURP was associated with a significantly better Qmax at 12 months compared to PAE. This was supported by a notable mean difference, despite high heterogeneity among the studies. Additionally, TURP led to a significant reduction in prostate volume, with moderate heterogeneity observed. These findings collectively suggest that while PAE and TURP offer comparable outcomes for symptom relief and quality of life, TURP might be more efficacious in improving certain functional parameters such as Qmax and prostate volume reduction at the 12-month follow-up.

While significant improvements were reported for both PAE and TURP treatment groups, at short-term follow-up they are more notable in the TURP groups. This could be indicative of the procedural differences. The rapid symptomatic relief observed at 3-month follow-up in TURP patients is due to the procedure's immediate resection and ablation of prostatic tissue, contrasting with the delayed onset of benefits from PAE, which relies on ischemic reduction of the prostate and may take up to 6 months to manifest [26, 27]. This difference underscores the distinct mechanisms of action between TURP's direct tissue removal, yielding prompt improvement in LUTS, and PAE's gradual prostate volume reduction, leading to a slower but progressive alleviation of symptoms [28].

Two studies reported significant improvements to IPSS and IPSS-QoL in the TURP group when compared with PAE at 3 months [21] and 24 months [19]. In addition, significant improvements to PV, PVR and Qmax were also reported at 3 months [19, 21] and at 24 months [19] in the TURP group. While PAE did appear to improve some outcome measures, most were not significant. When comparing PAE with OSP, Russo et al. [25] reported significant improvements in IPSS, IPSS-QoL, Qmax, PVR and PSA levels at 12 months in the OSP group. In the same study, PAE seemed to significantly improve erectile dysfunction at 6 and 12 months [25] although more studies are needed to confirm the true benefit of PAE compared to OSP.

Overall, fewer adverse events were noted for PAE, and some were reported in the TURP group but absent from the PAE group. These differences might be explained by the minimally invasive nature of PAE compared to TURP and provides promising insight into the safety of this procedure for BPH. Furthermore, more minor (Clavien Grade I and II) events were reported for PAE compared to some major events (Clavien Grade III and IV) for TURP. Similarly, when comparing PAE with OSP, Russo et al. reported a greater incidence of major adverse events in the OSP group [25]. These results corroborate those found in the literature which commonly report minor events after PAE such as hematospermia, urinary tract infections and dysuria [29]. This finding can likely be explained in part by the reduced risk of more major events such as bleeding complications due to the lack of direct tissue resection in PAE. A previous meta-analysis investigating PAE further reported that 99% of complications were reported as minor out of a 33% overall complication rate [30]. In addition, postoperative sexual dysfunction is more commonly reported in TURP patients, which has been associated with erectile nerve damage and heating from the electrode used [31]. Lower sexual dysfunction rates after PAE is an important finding as these complications significantly impact patients’ QOL and well-being.

As mentioned, both TURP and OSP involve prostatic tissue resection, resulting in more immediate symptom improvements compared to PAE. Accordingly, the included studies reflect an overall higher surgical reintervention rate in PAE groups compared to TURP. This outcome was reported by 3 of 6 studies comparing PAE with TURP [19, 20, 24]. The literature reports that approximately 20–36% of PAE patients experience clinical failure, commonly requiring subsequent intervention to promote symptom improvements [32, 33]. Rates of clinical failure can vary depending on the study population and inclusion criteria; however, factors with strong predictive power for PAE success or failure still remain unclear and require further study [34]. Additionally, differences in PAE techniques such as embolic particle sizes may lead to differences in success rates between studies. Embolic particle sizes ranged from 250—500 microns in the included studies. A meta-analysis examining the relationship between embolic particle size and PAE outcome determined that smaller embolic particles were associated with a more notable reduction in IPSS at 12-months [35]. However, it should be noted that the optimal particle size has yet to be determined and study protocols and outcomes for PAE remain quite varied.

Furthermore, reintervention and failure rates can be influenced by several factors. In our study, Abt et al. 2021 [19], observed that 25% of their cases underwent unilateral embolization, although no direct cause of failure was reported. In Carnevale’s study, 13.3% of patients in the oPAE group had unilateral embolization, attributed to severe atherosclerosis or occlusion of the IVA on one side [20]. According to Costa and colleagues [33], one crucial factor for procedural failure is the presence of non-target embolization, which can significantly impact the efficacy of the procedure. More specifically, the complexity of prostatic arterial anatomy and the occurrence of collateral formation can challenge the success of PAE and potentially lead to the need for reintervention [36]. Technical aspects also play a pivotal role in PAE success. Gao et al., reported clinical failure rates of 9.4% for PAE patients, with a technical failure rate of 5.3%, suggesting higher failure rates may be linked to varying levels of expertise among urologists and interventional radiologists [21]. Bilhim et al. highlighted the importance of procedural accuracy in PAE. The study indicates that precise catheterization and embolization of both prostatic arteries are crucial for reducing reintervention rates. Incomplete or inaccurate embolization can result in persistent symptoms, leading to the need for further treatment [37].

There is some concern about the potential long-term consequences of radiation exposure from PAE [38]. As reflected in the included studies, PAE involves a longer procedure time than TURP and OSP, with a mean radiation exposure time of approximately 20–58 min [39]. While PAE has been proven to be an effective treatment for LUTS associated with BPH, this procedure’s reliance on ionizing radiation presents a risk that has not been studied extensively yet [40]. However, the longer procedure time for PAE ranging from 84—144.8 min compared to 59—83.5 min for TURP in the present included studies has been found to be more favourable in reducing the risk of blood loss and other serious complications reported when directly resecting and ablating prostatic tissue [41].

Most of the included studies did not present long-term comparative data, except for two studies by Abt et al. [19] and Gao et al. [21] which evaluated PAE and TURP at 24 months. Future RCTs are needed to establish the comparative long-term effects and benefits of these procedures. However, according to a longer-term study by Pisco et al. which evaluated PAE outcomes up to 6.5 years, the clinical success rate has been reported to be about 76% for PAE [42]. Furthermore, a study by Carnevale et al. reported a 23% symptom recurrence at 72 months for PAE patients [43]. Abt et al. demonstrated that the reduction of PV was less pronounced after PAE than TURP after 24-month follow up [19].

The present work differs from previous published systematic reviews published with respect to treatment modalities, methodology, inclusion criteria, outcomes, and demographics. We have reported the most recent evidence published on the efficacy of PAE compared with surgical and minimally invasive options for BPH. We reported clinical outcomes which used validated tools (IIEF questionnaire and IPSS-QoL scale), contributing to the reliability and objectivity of these results.

The reported studies indicate that PAE might improve BPH symptoms and urodynamic measures; however, the long-term comparative effectiveness of these procedures is not clearly established. PAE stands as an effective minimally invasive procedure for males with moderate to severe BPH who might not be suitable candidates for surgery for reasons such as advanced age or comorbidities. Future long-term studies are needed to provide more evidence regarding the effectiveness and safety of PAE compared with other treatments for BPH.

### Limitations

Some of the studies did not report all of the outcomes included in this review which limits the comparison of the evidence. In addition, some studies did not report 95% CIs, so values were estimated using WebPlotDigitizer [44] and standard deviations were imputed for studies that did not report variance using a conservative formula (correlation coefficient of 1). Risk of bias was a concern for most of the included studies. Some of the RCTs were underpowered and included smaller sample sizes; did not clearly define methods for randomization; and confounding factors were not always accounted for. Study populations and designs differed, limiting the ability to effectively compare the studies. Further, most of the included studies lacked long-term follow-up aside from two studies by Abt et al. [19] and Gao et al. [21] and so the evidence regarding the prospective symptom improvement in the PAE group is uncertain.

Lastly, the heterogeneity noted in our meta-analysis, as evidenced by high I^2^ values for certain outcomes, points to the diversity in the included studies' designs, patient demographics, and intervention methods. To manage this, we utilized random-effects models, which account for variance among studies, thus providing a refined estimation of the effects that respects the heterogeneity of the data. A methodological strength of our analysis is the exclusive pooling studies from the same design measuring outcomes at the same time point. Non-randomized studies were addressed descriptively, due to the presence of only one study which precluded pooling. Should more non-randomized studies have been available, they would have been aggregated together. This careful selection enhances the robustness of our findings, although the heterogeneity observed reminds us to consider these results within the appropriate clinical contexts.

## Conclusion

This systematic review includes the available comparative evidence on the use of PAE for BPH. Only RCTs and observational studies comparing PAE with TURP or OSP were included. The collected data shows that PAE might improve symptomatic BPH-LUTS; reduce adverse events and major complications; and provide an alternative option for males with BPH who cannot undergo surgery or have failed medical therapy. Whether PAE is more effective than TURP and OSP remains uncertain, thus ongoing, longer-term studies will provide valuable evidence of the effectiveness and safety of PAE compared with other BPH treatment options.

### Supplementary Information

**Additional file 1: Table S1.** International prostatic symptom score (IPSS) for prostatic artery embolization (PAE) vs. transurethral resection of the prostate (TURP). **Table S2.** International prostate symptom score-quality of life (IPSS-QoL) for prostatic artery embolization (PAE) vs. transurethral resection of the prostate (TURP). **Table S3.** International index of erectile function (IIEF) for prostatic artery embolization (PAE) vs. transurethral resection of the prostate (TURP). **Table S4.** Peak urinary flow rate (Qmax) for prostatic artery embolization (PAE) vs. transurethral resection of the prostate (TURP).** Table S5.** Post-void residual volume (PVR) for prostatic artery embolization (PAE) vs. transurethral resection of the prostate (TURP). **Table S6.** Prostate volume (PV) for prostatic artery embolization (PAE) vs. transurethral resection of the prostate (TURP). **Table S7. **Prostate-specific antigen (PSA) levels for prostatic artery embolization (PAE) vs. transurethral resection of the prostate (TURP).** Table S8. **Detailed adverse events PAE vs TURP.

## Data Availability

The datasets used and/or analyzed during the current study are available from the corresponding author on reasonable request.

## References

[1] Canadian Cancer Society / Société canadienne du cancer. Benign prostatic hyperplasia (BPH) Canadian Cancer Society Available from: https://cancer.ca/en/cancer-information/cancer-types/prostate/what-is-prostate-cancer/benign-prostatic-hyperplasia. Cited 2023 Nov 26.

[2] Vasanwala FF, Wong MYC, Ho HSS, Foo KT (2017). Benign prostatic hyperplasia and male lower urinary symptoms A guide for family physicians. Asian J Urol.

[3] Chughtai B, Forde JC, Thomas DDM, Laor L, Hossack T, Woo HH (2016). Benign prostatic hyperplasia. Nat Rev Dis Primers.

[4] Jiang YL, Qian LJ (2019). Transurethral resection of the prostate versus prostatic artery embolization in the treatment of benign prostatic hyperplasia a meta-analysis. BMC Urol.

[5] Lepor H (2011). Medical treatment of benign prostatic hyperplasia. Rev Urol.

[6] Xia Z, Li J, Yang X, Jing H, Niu C, Li X (2021). Robotic-Assisted vs Open Simple Prostatectomy for Large Prostates A Meta-Analysis. Front Surg.

[7] Ferretti M, Phillips J (2015). Prostatectomy for benign prostate disease open laparoscopic and robotic techniques. Can J Urol.

[8] Miernik A, Gratzke C (2020). Current Treatment for Benign Prostatic Hyperplasia. Dtsch Arztebl Int.

[9] Pariser JJ, Packiam VT, Adamsky MA, Bales GT (2016). Trends in Simple Prostatectomy for Benign Prostatic Hyperplasia. Curr Urol Rep.

[10] Hashem E, Elsobky S, Khalifa M (2020). Prostate Artery Embolization for Benign Prostate Hyperplasia Review: Patient Selection Outcomes, and Technique. Semin Ultrasound CT MR.

[11] Moher D, Liberati A, Tetzlaff J, Altman DG, PRISMA Group (2010). Preferred reporting items for systematic reviews and meta-analyses: the PRISMA statement. Int J Surg.

[12] Higgins JPT, Altman DG, Gøtzsche PC, Jüni P, Moher D, Oxman AD (2011). The Cochrane Collaboration’s tool for assessing risk of bias in randomised trials. BMJ.

[13] Jüni P, Loke Y, Pigott T, Ramsay C, Regidor D, Rothstein H (2016). Risk of bias in non-randomized studies of interventions (ROBINS-I): detailed guidance. Br Med J.

[14] Barry MJ, Fowler FJ, O’leary MP, Bruskewitz RC, Holtgrewe HL, Mebust WK (2017). The American Urological Association Symptom Index for Benign Prostatic Hyperplasia. J Urol.

[15] Rosen RC, Riley A, Wagner G, Osterloh IH, Kirkpatrick J, Mishra A (1997). The international index of erectile function IIEF a multidimensional scale for assessment of erectile dysfunction. Urology.

[16] Higgins JPT (2019). Cochrane Handbook for Systematic Reviews of Interventions.

[17] GRADE handbook. Available from: https://training.cochrane.org/resource/grade-handbook. Cited 2023 Nov 26.

[18] Guyatt GH, Oxman AD, Vist GE, Kunz R, Falck-Ytter Y, Alonso-Coello P (2008). GRADE an emerging consensus on rating quality of evidence and strength of recommendations. BMJ.

[19] Abt D, Müllhaupt G, Hechelhammer L, Markart S, Güsewell S, Schmid HP (2021). Prostatic Artery Embolisation Versus Transurethral Resection of the Prostate for Benign Prostatic Hyperplasia: 2-yr Outcomes of a Randomised Open-label Single-centre. Trial Eur Urol.

[20] Carnevale FC, Iscaife A, Yoshinaga EM, Moreira AM, Antunes AA, Srougi M (2016). Transurethral Resection of the Prostate TURP Versus Original and PErFecTED Prostate Artery Embolization PAE Due to Benign Prostatic Hyperplasia BPH Preliminary Results of a Single Center Prospective Urodynamic-Controlled Analysis. Cardiovasc Intervent Radiol.

[21] Gao YA, Huang Y, Zhang R, Yang YD, Zhang Q, Hou M (2014). Benign Prostatic Hyperplasia Prostatic Arterial Embolization versus Transurethral Resection of the Prostate—A Prospective, Randomized and Controlled Clinical Trial. Radiology.

[22] Insausti I, Sáez de Ocáriz A, Galbete A, Capdevila F, Solchaga S, Giral P (2020). Randomized Comparison of Prostatic Artery Embolization versus Transurethral Resection of the Prostate for Treatment of Benign Prostatic Hyperplasia. J Vasc Interv Radiol.

[23] Radwan A, Farouk A, Higazy A, Samir YR, Tawfeek AM, Gamal MA (2020). Prostatic artery embolization versus transurethral resection of the prostate in management of benign prostatic hyperplasia. Prostate Int.

[24] Ray AF, Powell J, Speakman MJ, Longford NT, DasGupta R, Bryant T (2018). Efficacy and safety of prostate artery embolization for benign prostatic hyperplasia: an observational study and propensity-matched comparison with transurethral resection of the prostate the UK-ROPE study. BJU Int.

[25] Russo GI, Kurbatov D, Sansalone S, Lepetukhin A, Dubsky S, Sitkin I (2015). Prostatic Arterial Embolization vs Open Prostatectomy A 1-Year Matched-pair Analysis of Functional Outcomes and Morbidities. Urology.

[26] Maclean D, Maher B, Modi S, Harris M, Dyer J, Somani B (2017). Prostate artery embolization a new minimally invasive treatment for lower urinary tract symptoms secondary to prostate enlargement. Ther Adv Urol.

[27] Malling B, Røder MA, Brasso K, Forman J, Taudorf M, Lönn L (2019). Prostate artery embolisation for benign prostatic hyperplasia a systematic review and meta-analysis. Eur Radiol.

[28] Sapoval M, Thiounn N, Descazeaud A, Déan C, Ruffion A, Pagnoux G, et al. Prostatic artery embolisation versus medical treatment in patients with benign prostatic hyperplasia (PARTEM): a randomised, multicentre, open-label, phase 3, superiority trial. The Lancet Regional Health – Europe 2023 1;31. Available from: 10.1016/j.lanepe.2023.100672. 10.1016/j.lanepe.2023.100672 PMC10320610 37415648

[29] Naidu SG, Narayanan H, Saini G, Segaran N, Alzubaidi SJ, Patel IJ, et al. Prostate Artery Embolization-Review of Indications, Patient Selection, Techniques and Results. J Clin Med Res. 2021 10(21). Available from: 10.3390/jcm10215139. 10.3390/jcm10215139 PMC8584630 34768659

[30] Uflacker A, Haskal ZJ, Bilhim T, Patrie J, Huber T, Pisco JM (2016). Meta-Analysis of Prostatic Artery Embolization for Benign Prostatic Hyperplasia. J Vasc Interv Radiol.

[31] Manfredi C, García-Gómez B, Arcaniolo D, García-Rojo E, Crocerossa F, Autorino R (2022). Impact of Surgery for Benign Prostatic Hyperplasia on Sexual Function A Systematic Review and Meta-analysis of Erectile Function and Ejaculatory Function. Eur Urol Focus.

[32] Smith NA, Katz JE, Shah HN (2021). Enbloc laser enucleation of prostate after prostatic artery embolization. Urol Video J.

[33] Costa NV, Torres D, Pisco J, Pinheiro LC, Martins FE, Oliveira AG (2020). Repeat Prostatic Artery Embolization for Patients with Benign Prostatic Hyperplasia. J Vasc Interv Radiol.

[34] Sun F, Lucas-Cava V, Sánchez-Margallo FM (2020). Clinical predictive factors in prostatic artery embolization for symptomatic benign prostatic hyperplasia a comprehensive review. Transl Androl Urol.

[35] Geevarghese R, Harding J, Parsons N, Hutchinson C, Parsons C (2020). The relationship of embolic particle size to patient outcomes in prostate artery embolisation for benign prostatic hyperplasia: a systematic review and meta-regression. Clin Radiol.

[36] de Assis AM, Moreira AM, Carnevale FC (2019). Angiographic Findings during Repeat Prostatic Artery Embolization. J Vasc Interv Radiol.

[37] Bilhim T, Costa NV, Torres D (2021). Prostatic Artery Embolization for Benign Prostatic Hyperplasia A Primer for Interventional Radiologists. Arab J Int Radiol.

[38] Maclean D, Francis Bryant CT, Vigneswaran G, Bryant TJ, Harris M, Somani B (2022). Comprehensive Review on Current Controversies and Debate in Prostate Artery Embolization. Turk J Urol.

[39] Xiang P, Guan D, Du Z, Hao Y, Yan W, Wang Y (2021). Efficacy and safety of prostatic artery embolization for benign prostatic hyperplasia a systematic review and meta-analysis of randomized controlled trials. Eur Radiol.

[40] Liu J, Zhang K, Wu X, Cui B, Liang Z (2022). Radiation dose of prostatic artery embolization for benign prostatic hyperplasia: A protocol for systematic review. Medicine.

[41] Abt D, Hechelhammer L, Müllhaupt G, Markart S, Güsewell S, Kessler TM, Schmid HP, Engeler DS, Mordasini L. Comparison of prostatic artery embolisation (PAE) versus transurethral resection of the prostate (TURP) for benign prostatic hyperplasia: randomised, open label, non-inferiority trial. Bmj. 2018;361. 10.1136/bmj.k2338 PMC6006990 29921613

[42] Pisco JM, Bilhim T, Pinheiro LC, Fernandes L, Pereira J, Costa NV (2016). Medium- and Long-Term Outcome of Prostate Artery Embolization for Patients with Benign Prostatic Hyperplasia Results in 630 Patients. J Vasc Interv Radiol.

[43] Carnevale FC, Moreira AM, de Assis AM, Antunes AA, Cristina de Paula Rodrigues V, Srougi M (2020). Prostatic Artery Embolization for the Treatment of Lower Urinary Tract Symptoms Due to Benign Prostatic Hyperplasia 10 Years’ Experience. Radiology.

[44] Rohatgi A. WebPlotDigitizer - Extract data from plots, images, and maps Available from: https://automeris.io/WebPlotDigitizer/citation.html. Cited 2023 Nov 26.

