# Supplementary material for: Comparing prostatic artery embolization to surgical and minimally invasive procedures for the treatment of benign prostatic hyperplasia: a systematic review and meta-analysis
Source: BMC Urol. 2024 Jan 28;24:22. doi: 10.1186/s12894-023-01397-1 (PMC10822184; doi:10.1186/s12894-023-01397-1)
Supplement: Supplementary file 1 — Additional file 1: Table S1. International prostatic symptom score (IPSS) for prostatic artery embolization (PAE) vs. transurethral resection of the prostate (TURP). Table S2. International prostate symptom score-quality of life (IPSS-QoL) for prostatic artery embolization (PAE) vs. transurethral resection of the prostate (TURP). Table S3. International index of erectile function (IIEF) for prostatic artery embolization (PAE) vs. transurethral resection of the prostate (TURP). Table S4. Peak urinary flow rate (Qmax) for prostatic artery embolization (PAE) vs. transurethral resection of the prostate (TURP). Table S5. Post-void residual volume (PVR) for prostatic artery embolization (PAE) vs. transurethral resection of the prostate (TURP). Table S6. Prostate volume (PV) for prostatic artery embolization (PAE) vs. transurethral resection of the prostate (TURP). Table S7. Prostate-specific antigen (PSA) levels for prostatic artery embolization (PAE) vs. transurethral resection of the prostate (TURP). Table S8. Detailed adverse events PAE vs TURP. [file 12894_2023_1397_MOESM1_ESM.docx]

**Appendix**

**Table S1: International prostatic symptom score (IPSS) for prostatic artery embolization (PAE) vs. transurethral resection of the prostate (TURP)**

| **Mean IPSS (95% CI): Baseline** | | | | |
| --- | --- | --- | --- | --- |
| **Study** | **N** | **PAE** | **TURP** | **P Value** |
| Abt et al., 2021^9^ | PAE: 48  TURP: 51 | 19.4 (17.5–21.1) | 17.6 (15.9–19.2) | NR |
| Carnevale et al., 2016^10^ | Original PAE: 15  PErFecTED PAE: 15  TURP: 15 | Original: 25.3 (23.5–27.1)  PErFecTED: 24.6 (22.8–26.4) | 27.6 (26.0–29.2) | .08 |
| Gao et al., 2014^11^ | PAE: 54  TURP: 53 | 24.3 (17.9–30.7) | 24.7 (18.5–30.9) | NR |
| Insausti et al., 2020^12^ | PAE: 23  TURP: 22 | 26.6 (24.1–28.9) | 26.9 (23.8–29.9) | NR |
| Ray et al., 2018^13^ | PAE: 216  TURP: 89 | 21.3 (Median 22.0) | 21.6 (Median 22.0) | .926 |
| Radwan et al., 2020 [^23^](https://paperpile.com/c/Ywo9eZ/2Tli) | PAE: 20  TURP:40 | 26.7  (25.3 - 28.1) | 26.7 (25.5 - 27.9) | NR |
| **Mean IPSS (95% CI): 3 Months** | | | | |
| Abt et al., 2021^9^ | PAE: 48  TURP: 51 | 10.2 (8.2–12.1) | 6.8 (5.4–8.3) | .31 |
| Gao et al., 2014^11^ | PAE: 54  TURP: 53 | 15.6 (8.1-23.1) | 11.0 (6.5-15.6) | <.001 |
| Insausti et al., 2020^12^ | PAE: 23  TURP: 22 | 5.0 (1.7-6.5) | 12.6 (8.4-15.7) | NR |
| Ray et al., 2018^13^ | PAE: 159  TURP: 45 | 9.6 (Median 8.5) | 9.8 (Median 5.0) | NR |
| **Mean IPSS (95% CI): 6 Months** | | | | |
| Abt et al., 2021^9^ | PAE: 46  TURP: 51 | 10.2  (8.1-12.3) | 5.7  (4.3-7.1) | NR |
| Gao et al., 2014^11^ | PAE: 54  TURP: 53 | 12.8 (7.0-18.7) | 11.3 (6.4-16.3) | NS |
| Insausti et al., 2020^12^ | PAE: 23  TURP: 22 | 4.1 (1.6-5.0) | 10.2 (5.7-13.5) | NR |
| Ray et al., 2018^13^ | PAE: 133  TURP: 31 | 10.1 (Median 9.0) | 8.0 (Median 4.0) | NR |
| Radwan et al., 2020 [^23^](https://paperpile.com/c/Ywo9eZ/2Tli) | PAE: 20  TURP:40 | 12.7  (11.4-17.1) | 8.7  (7.6-11.1) | NR |
| **Mean IPSS (95% CI): 12 Months** | | | | |
| Abt et al., 2021^9^ | PAE: 40  TURP: 50 | 10.1  (7.7-12.5) | 5.8  (4.5-7.1) | NR |
| Carnevale et al., 2016^10^ | Original PAE: 15  PErFecTED PAE: 15  TURP: 15 | Original: 12.8 (8.8–16.8)  PErFecTED: 3.6 (2.1–5.1) | 6.1 (1.7–10.5) | Original PAE vs. TURP: .012  Original PAE vs. PErFecTED: < .001  PErFecTED vs. TURP: > .20 |
| Gao et al., 2014^11^ | PAE: 54  TURP: 53 | 10.9 (6.8–15.0) | 10.2 (5.9–14.5) | NS |
| Insausti et al., 2020^12^ | PAE: 23  TURP: 22 | 5.6 (2.4–7.1) | 8.8 (5.2–10.9) | NR |
| Ray et al., 2018^13^ | PAE: 132  TURP: 29 | 10.0 (Median 9.0) | 7.2 (Median 5.0) | NR |
| **Mean IPSS (95% CI): 24 Months** | | | | |
| Abt et al., 2021^9^ | PAE: 34  TURP: 47 | 9.7  (7.5-12.1) | 5.2  (4.3-6.3) | .05 |
| Gao et al., 2014^11^ | PAE: 47  TURP: 48 | 8.7 (4.6-12.8) | 8.4 (4.2-12.6) | NS |

Abbreviations: CI, confidence interval; NA, not applicable; NR, not reported; NS, not significant; PErFecTED, proximal embolization first then embolize distal method of PAE

**Table S2: International prostate symptom score-quality of life (IPSS-QoL) for prostatic artery embolization (PAE) vs. transurethral resection of the prostate (TURP)**

| **Mean IPSS-QoL (95% CI): Baseline** | | | | |
| --- | --- | --- | --- | --- |
| **Study** | **N** | **PAE** | **TURP** | **P Value** |
| Abt et al., 2021^9^ | PAE: 48  TURP: 51 | 4.0 (3.7–4.3) | 4.2 (3.9–4.5) | NR |
| Carnevale et al., 2016^10^ | Original PAE: 15  PErFecTED PAE: 15  TURP: 15 | Original: 4.7 (4.4–5.0)  PErFecTED: 4.7 (4.4–5.0) | 4.6 (4.2–5.0) | > .2 |
| Gao et al., 2014^11^ | PAE: 54  TURP: 53 | 4.8 (4.0–5.6) | 4.6 (3.9–5.3) | NS |
| Insausti et al., 2020^12^ | PAE: 23  TURP: 22 | 4.5 (4.1–4.9) | 4.7 (4.3–5.2) | NR |
| Ray et al., 2018^13^ | PAE: 189  TURP: 48 | 4.6 (Median 5.0) | 4.9 (Median 5.0) | .076 |
| **Mean IPSS-QoL (95% CI): 3 Months** | | | | |
| Abt et al., 2021^9^ | PAE: 48  TURP: 51 | 1.7 (1.2–2.1) | 1.6 (1.1–2.0) | .2 |
| Gao et al., 2014^11^ | PAE: 54  TURP: 53 | 2.9 (1.9–3.9) | 2.3 (1.3–3.3) | < .001 |
| Insausti et al., 2020^12^ | PAE: 23  TURP: 22 | 0.9 (0.5–1.3) | 2.1 (1.5–2.7) | NR |
| Ray et al., 2018^13^ | PAE: 160 TURP: 46 | 1.9 (Median 2.0) | 1.9 (Median 2.0) | NR |
| **Mean IPSS-QoL (95% CI): 6 Months** | | | | |
| Abt et al., 2021^9^ | PAE: 46  TURP: 51 | 1.6  (1.1-2.0) | 1.1  (0.7-1.5) | NR |
| Gao et al., 2014^11^ | PAE: 54  TURP: 53 | 2.2 (1.1–3.3) | 2.3 (1.4–3.2) | NS |
| Insausti et al., 2020^12^ | PAE: 23  TURP: 22 | 0.7 (0.3–1.0)a | 1.7 (1.0–2.3) | NR |
| Ray et al., 2018^13^ | PAE: 135 TURP: 35 | 2.1 (Median 2.0) | 1.9 (Median 1.0) | NR |
| **Mean IPSS-QoL (95% CI): 12 Months** | | | | |
| Abt et al., 2021^9^ | PAE: 40  TURP: 50 | 1.5  (1.1-2.0) | 0.9  (0.5-1.30 | NR |
| Carnevale et al., 2016^10^ | Original PAE: 15  PErFecTED PAE: 15  TURP: 15 | Original: 2.2 (1.6–2.8)  PErFecTED: 1.6 (1.2–2.0) | 0.9 (0.2–1.6) | NR |
| Gao et al., 2014^11^ | PAE: 54  TURP: 53 | 1.9 (1.0–2.8) | 1.8 (0.9–2.7) | NS |
| Insausti et al., 2020^12^ | PAE: 23  TURP: 22 | 0.7 (0.4–1.0) | 1.6 (1.2–2.1) | NR |
| Ray et al., 2018^13^ | PAE: 133  TURP: 31 | 2.0 (Median 2.0) | 1.5 (Median 1.0) | NR |
| **Mean IPSS-QoL (95% CI): 24 Months** | | | | |
| Abt et al., 2021^9^ | PAE: 34  TURP: 47 | 1.7  (1.2-2.2) | 1.0  (0.6-1.3) | .002 |
| Gao et al., 2014^11^ | PAE: 47  TURP: 48 | 1.6 (0.7–2.5) | 1.4 (0.6–2.2) | NS |

Abbreviations: CI, confidence interval; NA, not applicable; NR, not reported; NS, not significant; PErFecTED, proximal embolization first then embolize distal method of PAE.

**Table S3: International index of erectile function (IIEF) for prostatic artery embolization (PAE) vs. transurethral resection of the prostate (TURP)**

| **Mean IIEF-5 (95% CI): Baseline** | | | | |
| --- | --- | --- | --- | --- |
| **Study** | **N** | **PAE** | **TURP** | **P Value** |
| Abt et al., 2021^9^ | PAE: 48  TURP: 51 | 15.2 (12.9–17.4) | 13.1 (10.9–15.3) | NR |
| Carnevale et al., 2016^10^ | Original PAE: 15  PErFecTED PAE: 15  TURP: 15 | Original: 14.3 (10.9–17.7)  PErFecTED: 17.3 (14.6–20.0) | 12.5 (9.2–15.8) | .05 |
| Ray et al., 2018^13^ | PAE: 164  TURP: 36 | 14.4 (Median 15.0) | 14.4 (Median 15.0) | .906 |
| **Mean IIEF-5 (95% CI): 3 Months** | | | | |
| Abt et al., 2021^9^ | PAE: 48  TURP: 51 | 14.6 (12.0–17.2) | 11.7 (9.1–14.2) | .5 |
| Ray et al., 2018^13^ | PAE: 126 TURP: 28 | 16.2 (Median 18.0) | 15.6 (Median 16.0) | NR |
| **Mean IIEF-5 (95% CI): 6 Months** | | | | |
| Abt et al., 2021^9^ | PAE: 46  TURP: 51 | 15.2  (12.4-18.0) | 11.7  (9.0-14.3) | NR |
| Ray et al., 2018^13^ | PAE: 100 TURP: 20 | 17.0 (Median 19.0) | 19.2 (Median 20.0) | NR |
| **Mean IIEF-5 (95% CI): 12 Months** | | | | |
| Abt et al., 2021^9^ | PAE: 40  TURP: 50 | 14.5  (11.1-17.8) | 12.5  (9.9-15.1) | NR |
| Carnevale et al., 2016^10^ | Original PAE: 15  PErFecTED PAE: 15  TURP: 15 | Original: 12.6 (9.7–15.5)  PErFecTED: 18.7 (17.1–20.3) | 16.1 (13.2–19.0) | NR |
| Ray et al., 2018^13^ | PAE: 102  TURP: 20 | 16.3 (Median 19.0) | 14.8 (Median 13.5) | NR |
| **Mean IIEF-5 (95% CI): 24 Months** | | | | |
| Abt et al., 2021^9^ | PAE: 34  TURP: 47 | 14.4  (11.1-17.6) | 11.3  (8.7-13.8) | .6 |

Abbreviations: CI, confidence interval; NA, not applicable; NR, not reported; PErFecTED, proximal embolization first then embolize distal method of PAE.

**Table S4: Peak urinary flow rate (Qmax) for prostatic artery embolization (PAE) vs. transurethral resection of the prostate (TURP)**

| **Mean Qmax, mL/s (95% CI): Baseline** | | | | |
| --- | --- | --- | --- | --- |
| **Study** | **N** | **PAE** | **TURP** | **P Value** |
| Abt et al., 2021^9^ | PAE: 48  TURP: 51 | 7.5 (6.3–8.6) | 7.2 (6.1–8.5) | NR |
| Carnevale et al., 2016^10^ | Original PAE: 15  PErFecTED PAE: 15  TURP: 15 | Original: 7.0 (5.2–8.8)  PErFecTED: 5.1 (3.6–6.6) | 9.7 (7.8–11.6) | .004 |
| Gao et al., 2014^11^ | PAE: 54  TURP: 53 | 7.8 (5.3–10.3) | 7.3 (5.0–9.6)a | NS |
| Insausti et al., 2020^12^ | PAE: 23  TURP: 22 | 7.7 (6.6–8.8) | 7.0 (5.9–8.2)a | NR |
| Ray et al., 2018^13^ | PAE: 132  TURP: 39 | 8.8 (Median 8.0) | 10.4 (Median 10.0) | .095 |
| Radwan et al., 2020 [^23^](https://paperpile.com/c/Ywo9eZ/2Tli) | PAE: 20  TURP:40 | 9.8  (7.7- 11.9) | 9.8  (8.31 - 11.3) | NR |
| **Mean Qmax, mL/s (95% CI): 3 Months** | | | | |
| Abt et al., 2021^9^ | PAE: 48  TURP: 51 | 13.0 (11.4–14.8) | 22.5 (18.7–26.4) | <.001 |
| Gao et al., 2014^11^ | PAE: 54  TURP: 53 | 17.3 (13.6–21.4) | 21.4 (16.6–26.2) | <.001 |
| Insausti et al., 2020^12^ | PAE: 23  TURP: 22 | 12.3 (10.6–14.5) | 14.0 (10.9–18.4) | NR |
| Ray et al., 2018^13^ | PAE: 115 TURP: 21 | 13.6 (Median 12.0) | 20.8 (Median 19.0) | NR |
| **Mean Qmax, mL/s (95% CI): 6 Months** | | | | |
| Abt et al., 2021^9^ | PAE: 46  TURP: 51 | 13.4  (11.1-15.9) | 19.6  (16.0-23.1) | NR |
| Gao et al., 2014^11^ | PAE: 54  TURP: 53 | 21.5 (17.3–25.7) | 23.7 (20.4–27.0) | NS |
| Insausti et al., 2020^12^ | PAE: 23  TURP: 22 | 13.5 (11.9–15.3)a | 13.3 (9.6–16.2) | NR |
| Radwan et al., 2020 [^23^](https://paperpile.com/c/Ywo9eZ/2Tli) | PAE: 20  TURP:40 | 12.8  (10.7 - 14.9) | 16.8  (15.3 - 18.3) | NR |
| **Mean Qmax, mL/s (95% CI): 12 Months** | | | | |
| Abt et al., 2021^9^ | PAE: 40  TURP: 50 | 13.3  (10.6-16.1) | 17.7  (14.8-20.7) | NR |
| Carnevale et al., 2016^10^ | Original PAE: 15  PErFecTED PAE: 15  TURP: 15 | Original: 10.1 (6.8–13.4) PErFecTED: 8.4 (12.4–21.0) | 27.1 (22.7–31.5) | NR |
| Gao et al., 2014^11^ | PAE: 54  TURP: 53 | 22.1 (18.6–25.6) | 23.1 (19.9–26.4)a | NS |
| Insausti et al., 2020^12^ | PAE: 23  TURP: 22 | 13.8 (11.9–14.5) | 16.7 (12.2–19.2) | NR |
| Ray et al., 2018^13^ | PAE: 106 TURP: 13 | 14.1 (Median 13.5) | 22.3 (Median 20.0) | NR |
| **Mean Qmax, mL/s (95% CI): 24 Months** | | | | |
| Abt et al., 2021^9^ | PAE: 34  TURP: 47 | 11.6  (10.0-13.4) | 17.9  (15.1-21.0) | .001 |
| Gao et al., 2014^11^ | PAE: 47  TURP: 48 | 21.5 (17.9–25.1) | 22.1 (18.6–25.6) | NS |

Abbreviations: CI, confidence interval; NA, not applicable; NR, not reported; NS, not significant; PErFecTED, proximal embolization first then embolize distal method of PAE.

**Table S5: Post-void residual volume (PVR) for prostatic artery embolization (PAE) vs. transurethral resection of the prostate (TURP)**

| **Mean PVR, mL (95% CI): Baseline** | | | | |
| --- | --- | --- | --- | --- |
| **Study** | **N** | **PAE** | **TURP** | **P Value** |
| Abt et al., 2021^9^ | PAE: 48  TURP: 51 | 168.5 (116.9–220.3) | 230.7 (172.1–289.3) | NR |
| Carnevale et al., 2016^10^ | Original PAE: 15  PErFecTED PAE: 15  TURP: 15 | Original: 127.0 (76.4–177.6)  PErFecTED: 74.2 (49.3–99.1) | 78.3 (41.2–115.4) | > .2 |
| Gao et al., 2014^11^ | PAE: 54  TURP: 53 | 126.9 (57.9–195.7) | 115.4 (46.3–184.4) | NS |
| Insausti et al., 2020^12^ | PAE: 23  TURP: 22 | 82.3 (0.5–164.8) | 124.4 (55.0–194.6) | NS |
| Ray et al., 2018^13^ | PAE: 125  TURP: 46 | 161.6 (Median 130.0) | 263.6 (Median 204.0) | .004 |
| **Mean PVR, mL (95% CI): 3 Months** | | | | |
| Abt et al., 2021^9^ | PAE: 48  TURP: 51 | 70.3 (43.4–97.7) | 33.7 (21.1–45.9) | .003 |
| Gao et al., 2014^11^ | PAE: 54  TURP: 53 | 56.8 (17.4–95.8) | 33.2 (6.5–56.8) | .012 |
| Insausti et al., 2020^12^ | PAE: 23  TURP: 22 | 21.1 (10.2–33.0) | 22.6 (14.7–31.2) | NR |
| Ray et al., 2018^13^ | PAE: 110 TURP: 20 | 126.2 (Median 97.0) | 88.8 (Median 56.5) | NR |
| **Mean PVR, mL (95% CI): 6 Months** | | | | |
| Abt et al., 2021^9^ | PAE: 46  TURP: 51 | 60.7  (31.2-90.5) | 30.7  (16.6-45.1) | NR |
| Gao et al., 2014^11^ | PAE: 54  TURP: 53 | 39.2 (9.0–69.1) | 30.9 (5.4–56.1) | NS |
| Insausti et al., 2020^12^ | PAE: 23  TURP: 22 | 15.1 (7.8–23.4) | 18.9 (8.0–30.8) | NR |
| **Mean PVR, mL (95% CI): 12 Months** | | | | |
| Abt et al., 2021^9^ | PAE: 40  TURP: 50 | 53.5  (22.5-84.6) | 23.6  (11.8-35.3) | NR |
| Carnevale et al., 2016^10^ | Original PAE: 15  PErFecTED PAE: 15  TURP: 15 | Original: 62.3 (26.4–98.2) PErFecTED: 48.6 (15.4–81.8) | 8.3 (2.3–14.3) | NR |
| Gao et al., 2014^11^ | PAE: 54  TURP: 53 | 27.3 (3.3–51.0) | 22.3 (4.7–39.8) | NS |
| Insausti et al,. 2020^12^ | PAE: 23  TURP: 22 | 13.1 (4.9–22.3) | 15.0 (10.2–20.9) | NR |
| Ray et al., 2018^13^ | PAE: 101 TURP: 13 | 129.6 (Median 120.0) | 80.6 (Median 48.0) | NR |
| **Mean PVR, mL (95% CI): 24 Months** | | | | |
| Abt et al., 2021^9^ | PAE: 34  TURP: 47 | 91.6  (49.2-134.1) | 28.1  (13.0-43.5) | .005 |
| Gao et al., 2014^11^ | PAE: 47  TURP: 48 | 19.4 (4.0–34.4) | 15.2 (2.2–27.8) | NS |

Abbreviations: CI, confidence interval; NA, not applicable; NR, not reported; NS, not significant; PErFecTED, proximal embolization first then embolize distal method of PAE.

**Table S6: Prostate volume (PV) for prostatic artery embolization (PAE) vs. transurethral resection of the prostate (TURP)**

| **Mean PV, mL (95% CI): Baseline** | | | | |
| --- | --- | --- | --- | --- |
| **Study** | **N** | **PAE** | **TURP** | **P Value** |
| Abt et al., 2021^9^ | PAE: 48  TURP: 51 | 52.8 (43.8–61.8) | 56.5 (48.0–65.0) | NR |
| Carnevale et al., 2016^10^ | Original PAE: 15  PErFecTED PAE: 15  TURP: 15 | Original: 56.6 (45.7–67.5)  PErFecTED: 66.2 (59.8–72.6) | 63.0 (54.0–72.0) | > .2 |
| Gao et al., 2014^11^ | PAE: 54  TURP: 53 | 64.7 (44.8–84.2) | 63.5 (44.8–81.9) | NS |
| Insausti et al., 2020^12^ | PAE: 23  TURP: 22 | 60.0 (51.3–68.7) | 62.9 (52.9–72.8) | NR |
| Ray et al., 2018^13^ | PAE: 209  TURP: 28 | 101.2 (Median 89.0) | 65.6 (Median 58.5) | < .01 |
| Radwan et al., 2020 [^23^](https://paperpile.com/c/Ywo9eZ/2Tli) | PAE: 20  TURP:40 | 60  (50.3- 69.7) | 63.5  (56.5 - 70.5) | NR |
| **Mean PV, mL (95% CI): 3 Months** | | | | |
| Abt et al., 2021^9^ | PAE: 48  TURP: 51 | 40.7 (34.0–47.3) | 27.2 (22.7–31.7) | < .001 |
| Gao et al., 2014^11^ | PAE: 54  TURP: 53 | 43.4 (25.8–60.6) | 27.3 (14.5–39.8) | < .001 |
| Insausti et al., 2020^12^ | PAE: 23  TURP: 22 | 40.1 (33.4–47.0) | 21.7 (18.5–25.0) | NR |
| Ray et al., 2018^13^ | PAE: 192 TURP: 3 | 72.1 (Median 60.0) | 58.7 (Median 49.0) | NR |
| **Mean PV, mL (95% CI): 6 Months** | | | | |
| Gao et al., 2014^11^ | PAE: 54  TURP: 53 | 36.3 (23.1–49.4) | 26.8 (15.2–38.2)a | < .001 |
| Insausti et al., 2020^12^ | PAE: 23  TURP: 22 | 37.7 (31.3–44.1)a | 19.9 (16.4–23.7)a | NR |
| Radwan et al., 2020 [^23^](https://paperpile.com/c/Ywo9eZ/2Tli) | PAE: 20  TURP:40 | 49  (39.3 - 58.7) | 32.5  (25.5 - 39.5) | <0.001 |
| **Mean PV, mL (95% CI): 12 Months** | | | | |
| Carnevale et al., 2016^10^ | Original PAE: 15  PErFecTED PAE: 15  TURP: 15 | Original: 50.9 (41.3–60.5) PErFecTED: 50.0 (43.0–57.0) | 32.0 (26.2–37.8) | NR |
| Gao et al., 2014^11^ | PAE: 54  TURP: 53 | 35.6 (22.6–48.4) | 26.4 (15.6–36.8)a | < .001 |
| Insausti et al., 2020^12^ | PAE: 23  TURP: 22 | 39.5 (31.7–47.4) | 18.2 (15.2–21.2) | NR |
| Ray et al., 2018^13^ | PAE: 166 TURP: 0 | 72.8 (Median 58.0) | NA | NA |
| **Mean PV, mL (95% CI): 24 Months** | | | | |
| Abt et al., 2021^9^ | PAE: 34 TURP: 47 | 46.7  (36.3-56.9) | 30.2  (24.0-36.4) | .002 |
| Gao et al., 2014^11^ | PAE: 47  TURP: 48 | 34.9 (21.1–48.4)a | 26.6 (16.1–36.8)a | < .001 |

Abbreviations: CI, confidence interval; NA, not applicable; NR, not reported; NS, not significant; PErFecTED, proximal embolization first then embolize distal method of PAE.

**Table S7: Prostate-specific antigen (PSA) levels for prostatic artery embolization (PAE) vs. transurethral resection of the prostate (TURP)**

| **Mean PSA, ng/mL (95% CI): Baseline** | | | | |
| --- | --- | --- | --- | --- |
| **Study** | **N** | **PAE** | **TURP** | **P Value** |
| Abt et al., 2021^9^ | PAE: 48  TURP: 51 | 4.2 (2.4–5.3) | 4.5 (2.7–5.6) | NR |
| Carnevale et al., 2016^10^ | Original PAE: 15  PErFecTED PAE: 15  TURP: 15 | Original: 3.4 (2.3–4.5)  PErFecTED: 3.7 (2.6–4.8) | 3.2 (1.9–4.5) | > .2 |
| Gao et al., 2014^11^ | PAE: 54  TURP: 53 | 3.7 (1.7–5.7) | 3.6 (1.7–5.5) | NS |
| Insausti et al., 2020^12^ | PAE: 23  TURP: 22 | 3.5 (2.4–4.7) | 4.4 (0.8–8.0) | NR |
| **Mean PSA, ng/mL (95% CI): 3 Months** | | | | |
| Abt et al., 2021^9^ | PAE: 48  TURP: 51 | 2.3  (1.3-2.9) | 1.3  (0.9-1.5) | .07 |
| Gao et al., 2014^11^ | PAE: 54  TURP: 53 | 2.2 (1.3–3.1) | 1.5 (0.7–2.3) | .001 |
| Insausti et al., 2020^12^ | PAE: 23  TURP: 22 | 1.9 (1.3–2.6) | 0.9 (0.6–1.3) | NR |
| **Mean PSA, ng/mL (95% CI): 6 Months** | | | | |
| Abt et al., 2021^9^ | PAE: 46  TURP: 51 | 5.6  (1.3-3.4) | 1.5  (1.0-1.8) | NR |
| Gao et al., 2014^11^ | PAE: 57  TURP: 57 | 2.0 (1.2–2.8) | 1.7 (1.0–2.4) | NS |
| **Mean PSA, ng/mL (95% CI): 12 Months** | | | | |
| Abt et al., 2021^9^ | PAE: 40  TURP: 50 | 2.5  (1.1-3.4) | 1.8  (1.2-2.2) | NR |
| Carnevale et al., 2016^10^ | Original PAE: 15  PErFecTED PAE: 15  TURP: 15 | Original: 2.2 (1.6–2.8) PErFecTED: 1.7 (1.1–2.3) | 1.6 (1.1–2.1) | NS |
| Gao et al., 2014^11^ | PAE: 54  TURP: 53 | 2.1 (1.2–3.0)a | 1.6 (0.8–2.4)a | .009 |
| Insausti et al., 2020^12^ | PAE: 23  TURP: 22 | 2.8 (2.0–3.7) | 1.7 (0.9–2.4) | .013 |
| **Mean PSA, ng/mL (95% CI): 24 Months** | | | | |
| Abt et al., 2021^9^ | PAE: 34  TURP: 47 | 2.5  (1.4-3.2) | 2.0  (1.3-2.4) | .05 |
| Gao et al., 2014^11^ | PAE: 47  TURP: 48 | 2.1 (1.4–2.8) | 1.7 (0.9–2.5)a | .012 |

Abbreviations: CI, confidence interval; NA, not applicable; NR, not reported; NS, not significant.

**Table S8: Detailed adverse events PAE vs TURP**

| **Adverse Event** | **Abt et al., 2021** [^19^](https://paperpile.com/c/Ywo9eZ/hYpW) | | **Carnevale et al., 2016** [^20^](https://paperpile.com/c/Ywo9eZ/RWo1) | | | **Gao et al., 2014** [^21^](https://paperpile.com/c/Ywo9eZ/MTT7) | | **Insausti et al., 2020** [^22^](https://paperpile.com/c/Ywo9eZ/MVVX) | | **Radwan et al., 2020** [^23^](https://paperpile.com/c/Ywo9eZ/2Tli) | | **Ray et al., 2018** [^24^](https://paperpile.com/c/Ywo9eZ/02pk) | |
| --- | --- | --- | --- | --- | --- | --- | --- | --- | --- | --- | --- | --- | --- |
|  | **PAE**  **n (%)** | **TURP**  **n (%)** | **Original PAE**  **n (%)** | **PErFecTED PAE**  **n (%)** | **TURP**  **n (%)** | **PAE**  **n (%)** | **TURP**  **n (%)** | **PAE**  **n (%)** | **TURP**  **n (%)** | **PAE**  **n (%)** | **TURP**  **n (%)** | **PAE**  **n (%)** | **TURP**  **n (%)** |
| Bladder neck stenosis | 0/34  (0%) | 2/47  (4.3%) | NR | NR | NR | 0/47 (0%) | 1/48 (2.1%) | NR | NR | NR | NR | NR | NR |
| Bladder or urethral stricture | 0/34  (0%) | 3/47  (6.4%) | NR | NR | NR | 0/47 (0%) | 1/48 (2.1%) | 0/23 (0%) | 2/22 (9.1%) | NR | NR | NR | NR |
| Blood transfusion | NR | NR | NR | NR | NR | 0/54 (0%) | 2/53 (3.8%) | NR | NR | NR | NR | NR | NR |
| Clot retention | NR | NR | NR | NR | NR | 0/54 (0%) | 1/53 (1.9%) | NR | NR | NR | NR | NR | NR |
| Hematospermia | NR | NR | 1/15 (6.7%) | 1/15 (6.7%) | NR | NR | NR | NR | NR | NR | NR | 25/199 (12.6%) | 1/61 (1.6%) |
| Hematuria | 4/34  (11.8%) | 11/47  (23.4%) | 2/15 (13.3%) | 0/15 (0%) | 15/15 (100%) | 0/54 (0%) | 4/53 (7.5%) | 1/23 (4.3%) | 8/22 (36.4%) | NR | NR | 37/199 (18.6%) | 39/61 (63.9%) |
| Ejaculation disorders | NR | NR | 2/15 (13.3%) | 1/15 (6.7%) | 15/15  (100%) | NR | NR | 1/23 (4.3%) | 9/22 (40.9%) | NR | NR | 48/199 (24.1%) | 29/61 (47.5%) |
| Erectile dysfunction | NR | NR | NR | NR | NR | NR | NR | 1/23 (4.3%) | 5/22 (22.7%) | NR | NR | NR | NR |
| Left venous sinus damage | NR | NR | 0/15 (0%) | 0/15 (0%) | 1/15 (6.7%) | NR | NR | NR | NR | NR | NR | NR | NR |
| Rectal ischemia | NR | NR | NR | NR | NR | NR | NR | 1/23 (4.3%) | 0/22 (0%) | NR | NR | NR | NR |
| Transient rectal bleeding | NR | NR | 1/15 (6.7%) | 1/15 (6.7%) | 0/15 (0%) | NR | NR | NR | NR | NR | NR | NR | NR |
| Transient pubic bone ischemia | NR | NR | 1/15 (6.7%) | 0/15 (0%) | 0/15 (0%) | NR | NR | NR | NR | NR | NR | NR | NR |
| TUR syndrome | NR | NR | NR | NR | NR | 0/54 (0%) | 1/53 (1.9%) | NR | NR | NR | NR | NR | NR |
| Urinary incontinence | 0/34  (0%) | 3/47  (6.4%) | NR | NR | 4/15 (26.7%) | NR | NR | 0/23 (0%) | 3/22 (13.6%) | NR | NR | 2/199 (1.0%) | 2/61 (3.3%) |
| Urinary infection | 14/34  (41.2%) | 19/47  (40.4%) | NR | NR | NR | 1/54 (1.9%) | 2/53 (3.8%) | 0/23 (0%) | 4/22 (18.2%) | NR | NR | 10/199 (5.0%) | 1/61 (1.6%) |
| Urinary retention | 2/34  (5.9%) | 3/47  (6.4%) | NR | NR | NR | 14/54 (25.9%) | 3/53 (5.7%) | 5/23 (21.7%) | 4/22 (18.2%) | 2/20 | 0/40 | NR | NR |
| Other | 7/34  (20.6%) | 8/47  (17%) | NR | NR | NR | 22/53 (40.7%) | 13/53 (24.5%) | NR | NR | 4/20 | 1/40 | NR | NR |

Abbreviations: NR, not reported; PErFecTED, Proximal embolization first then embolize distal method of PAE; TUR, transurethral resection.
